# *Pandarus* Leach, 1816 (Copepoda: Siphonostomatoida: Pandaridae) species collected from elasmobranchs off South Africa with the description of *Pandarus echinifer* n. sp.

**DOI:** 10.1007/s11230-024-10167-y

**Published:** 2024-06-22

**Authors:** S. M. Dippenaar

**Affiliations:** Department of Biodiversity, University of Limpopo, Private Bag X1106, Sovenga, 0727 South Africa

## Abstract

Eight species of *Pandarus* Leach, 1816 collected from hosts caught off South Africa are reported. These species include *P*. *bicolor* Leach, 1816, *P*. *niger* Kirtisinghe, 1950 and *P*. *carcharhini* Ho, 1963 belonging to the “bicolor” group and *P*. *cranchii* Leach, 1819, *P*. *satyrus* Dana, 1849, *P*. *smithii* Rathbun, 1886 and *P*. *sinuatus* Say, 1818 belonging to the “cranchii” group. Notes on previous and new distinguishing features are provided with illustrations, specifically the relative lengths of the dorsal plates and caudal rami as well as the structure of the distomedial spine on the second segment of leg 1 exopod. Additionally, illustrated re-descriptions are provided for *P*. *satyrus* and *P*. *sinuatus*. Furthermore, a new species *Pandarus echinifer*** n. sp.**, also belonging to the “cranchii” group, collected from the snaggletooth shark *Hemipristis elongata* (Klunzinger) is described. This species is most similar to *P*. *sinuatus* but can be distinguished from it by the heavily spinulated distomedial spine on the last segment of the first leg exopod. Molecular analysis of the cytochrome oxidase I partial gene is used to calculate sequence divergences amongst different individuals and species. According to the results (as well as based on morphological characters) *P*. *rhincodonicus* Norman, Newbound & Knott, 2000 is a synonym of *P*. *cranchii*. New hosts and geographic localities from South Africa (and Ningaloo Park, Western Australia) are reported.

## Introduction

**Fig. 1:**
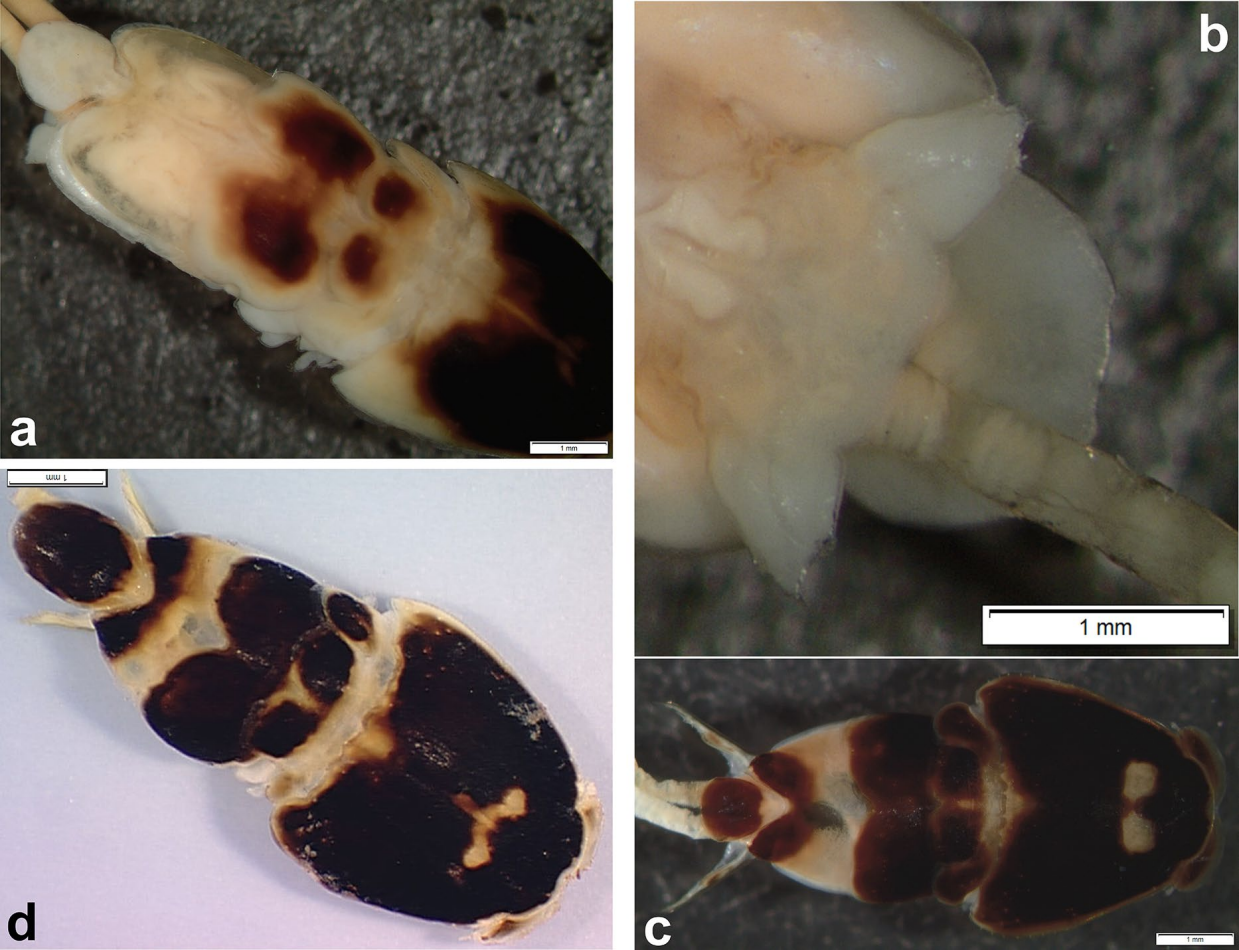
*Pandarus* species females of the “bicolor” group, adult female. a, *Pandarus bicolor*, Leach, 1816; b, Abdomen and caudal rami, ventral view; c, *Pandarus niger* Kirtisinghe, 1950; d, *Pandarus carcharhini* Ho, 1963.

**Fig. 2:**
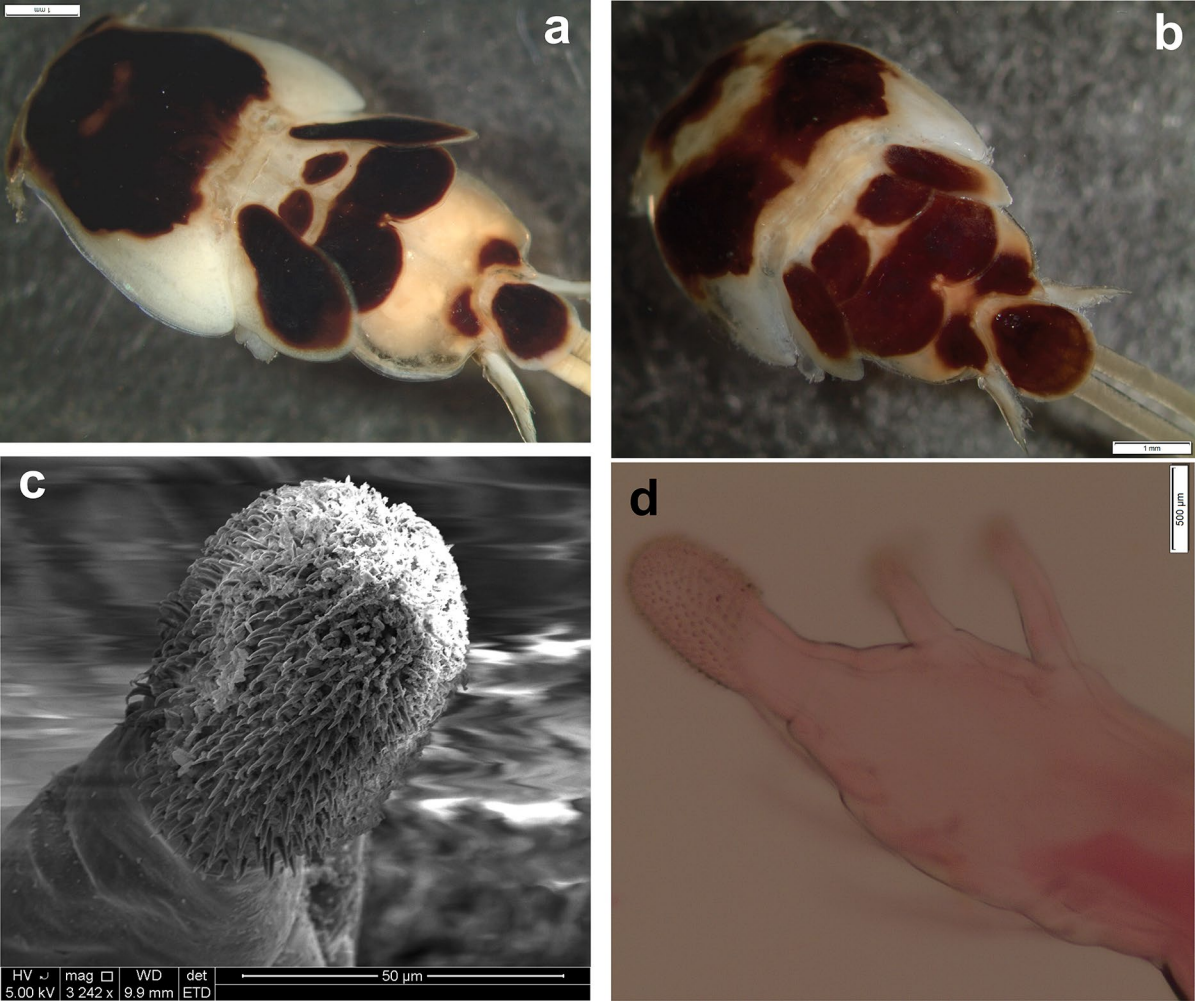
*Pandarus cranchii* Leach, 1819. a, adult female from *Sphyrna lewini* (Griffith & Smith); b, adult female from *Rhincodon typus* Smith; c, scanning electron micrograph of distomedial spinulated process of leg 1 exopod distal segment; d, light microscope photo of distomedial spinulated process of leg 1 exopod distal segment.

**Fig. 3:**
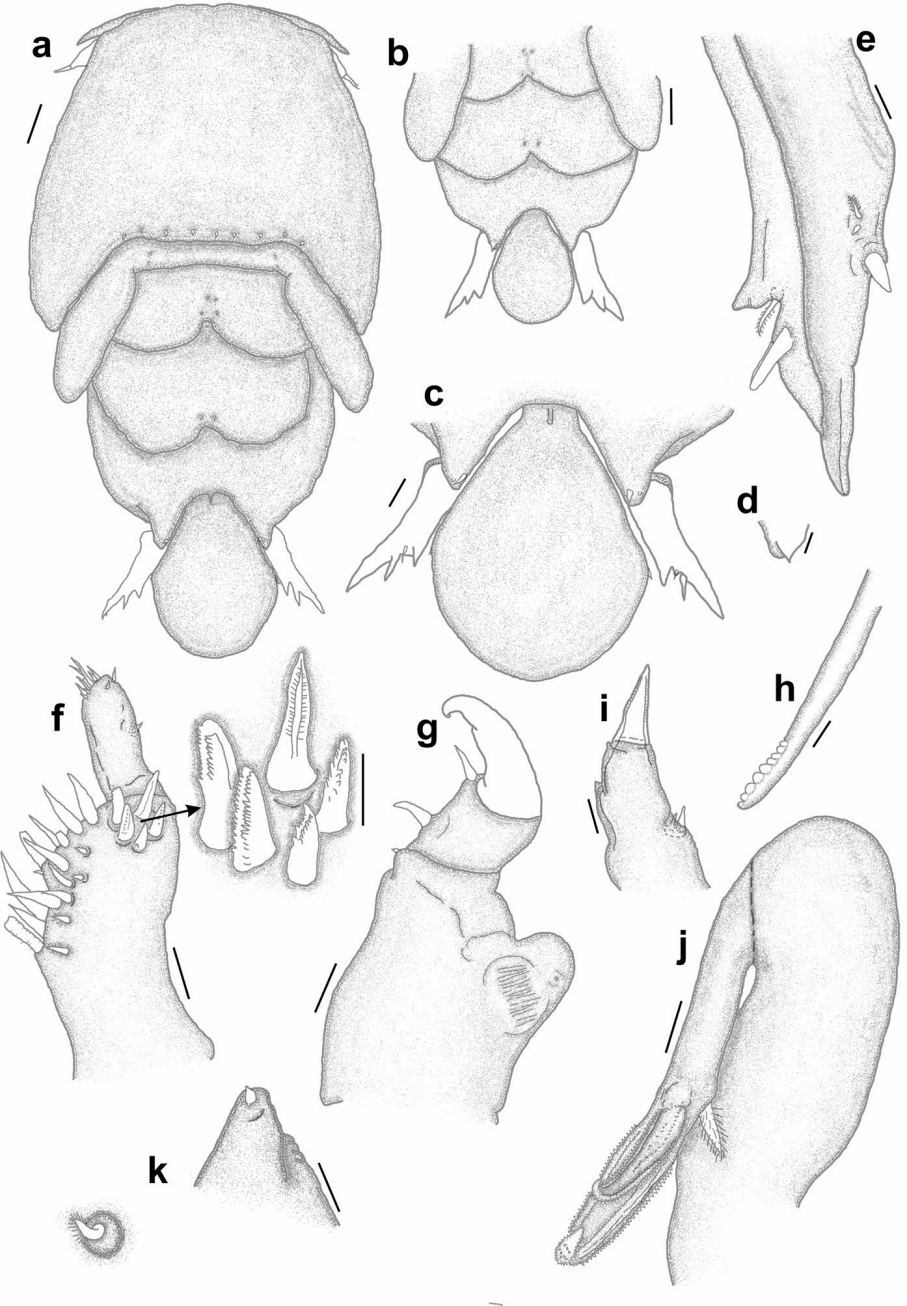
*Pandarus satyrus* Dana, 1849. a, adult female; b. posterior part of another specimen indicating lengths of dorsal plates and caudal rami; c, posterior part of genital complex, abdominal plate and caudal rami; d, posterior protrusion of genital complex with spine; e, distal part of caudal ramus; f, antennule, with enlarged setae (50 µm); g, antenna; h, mandible; i, maxillule; j, maxilla; k, leg 5. Scale-bars: a, b, 0.5 mm; c, 0.2 mm; d, 0.1 mm; e, f, g, j 100 µm; h, 10 µm; i, k, 50 µm.

**Fig. 4:**
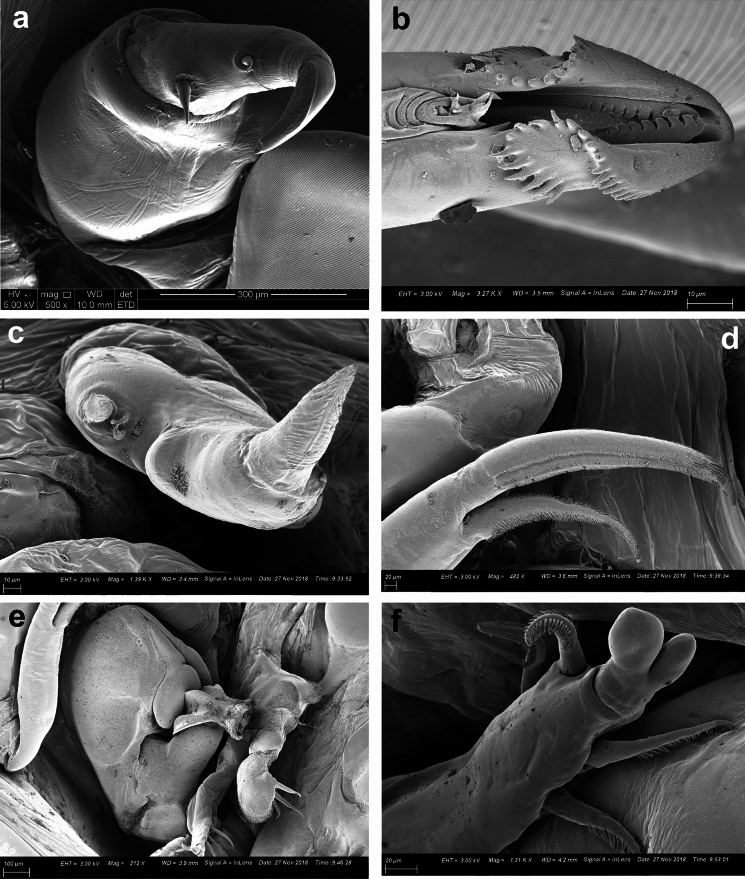
*Pandarus satyrus* Dana, 1849 scanning electron micrographs. a, antenna; b, distal part of oral cone with mandibles; c, maxillule; d, distal part of maxilla with calamus and canna; e, maxilliped attached to host scale; f, distomedial bifid process of leg 1 exopod distal segment.

**Fig. 5:**
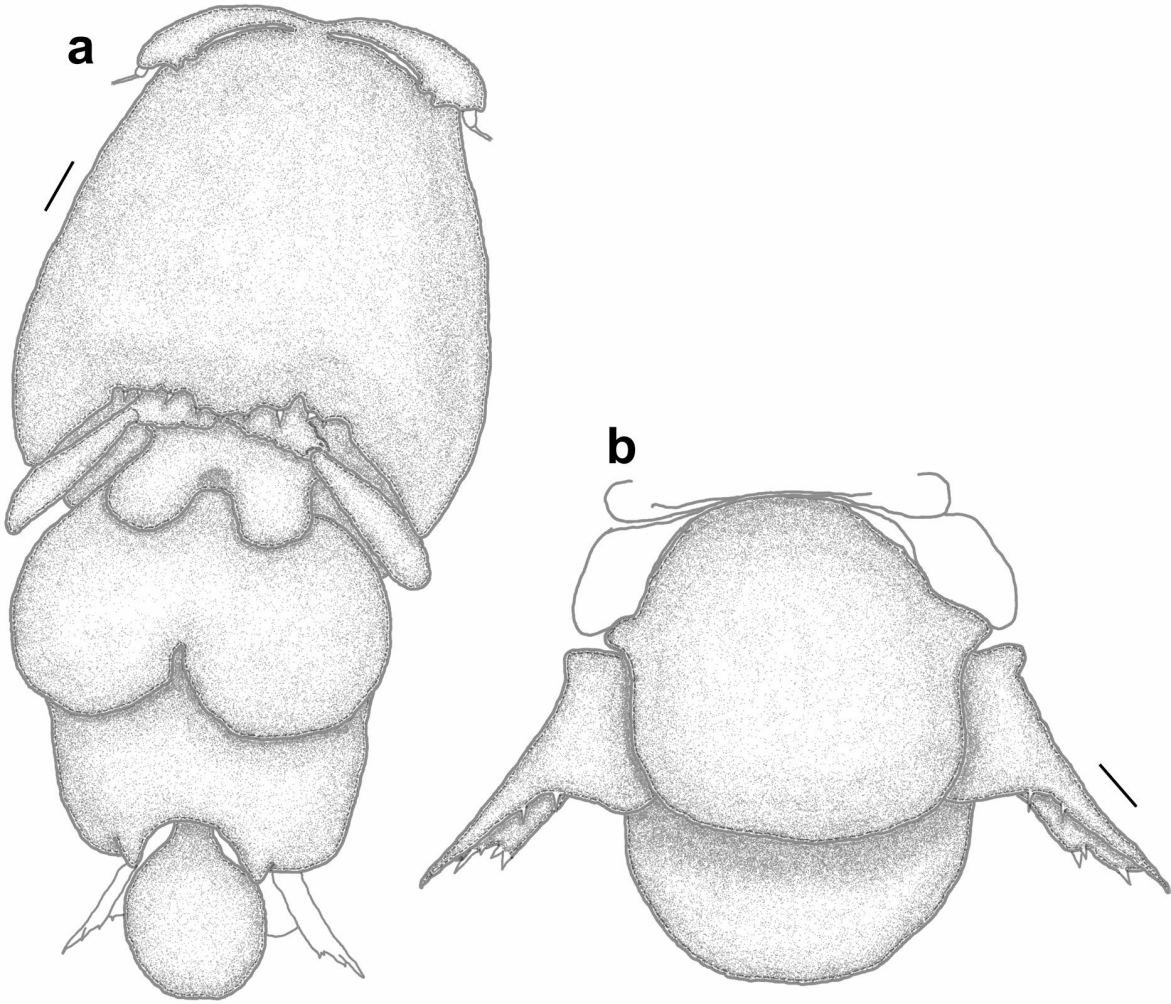
*Pandarus smithii* Rathbun, 1886. a, adult female; b, abdomen and caudal rami, ventral view. Scale-bars: a, 0.5 mm; b, 0.2 mm.

**Fig. 6:**
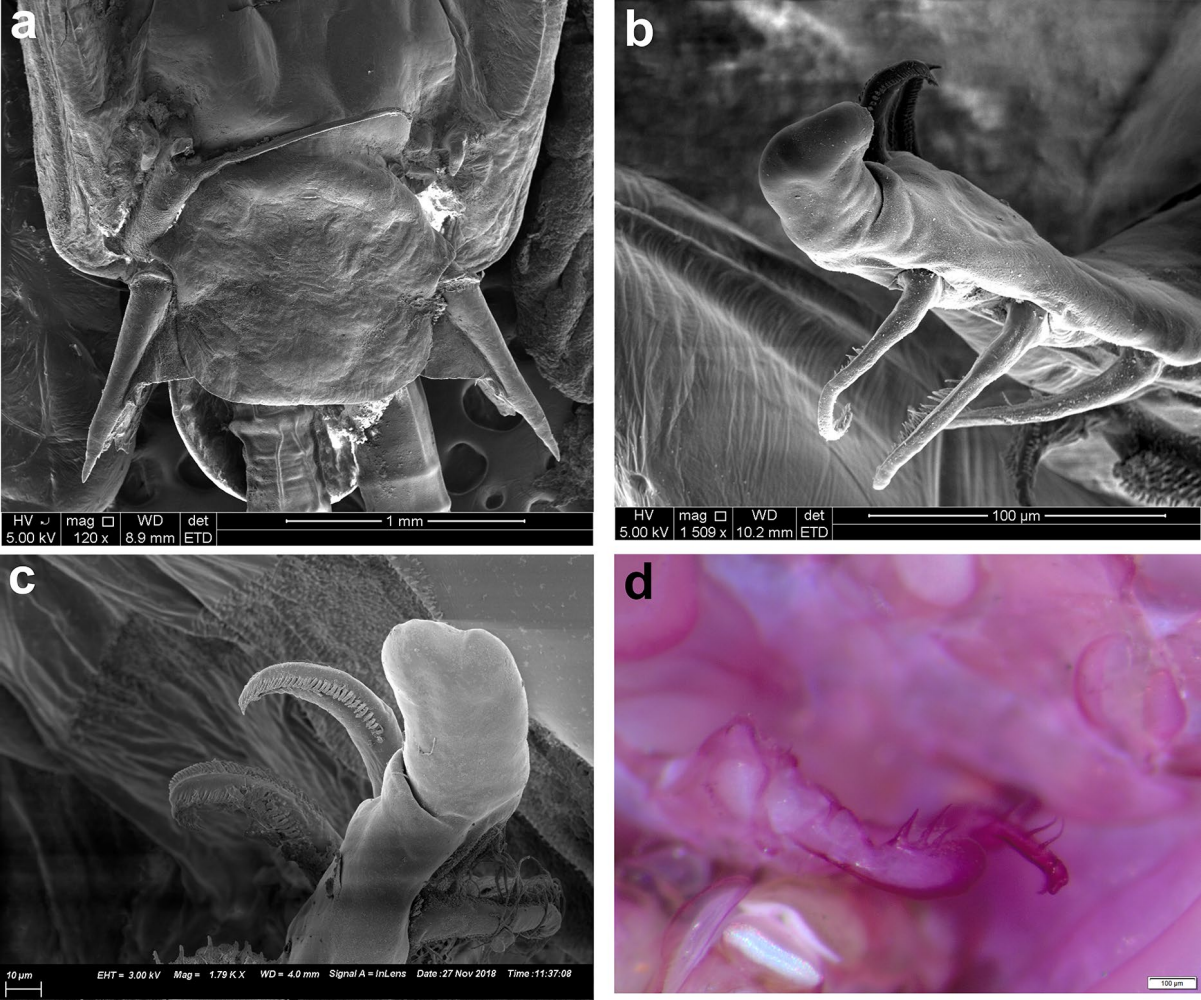
*Pandarus smithii* Rathbun, 1886, scanning electron micrographs. a, abdomen and caudal rami, ventral view; b, smooth distomedial process with a slightly extended tip of leg 1 exopod distal segment; c, smooth distomedial process with a slightly extended tip of leg 1 exopod (different view); d, light microscope photo of distomedial smooth process of leg 1 exopod.

**Fig. 7:**
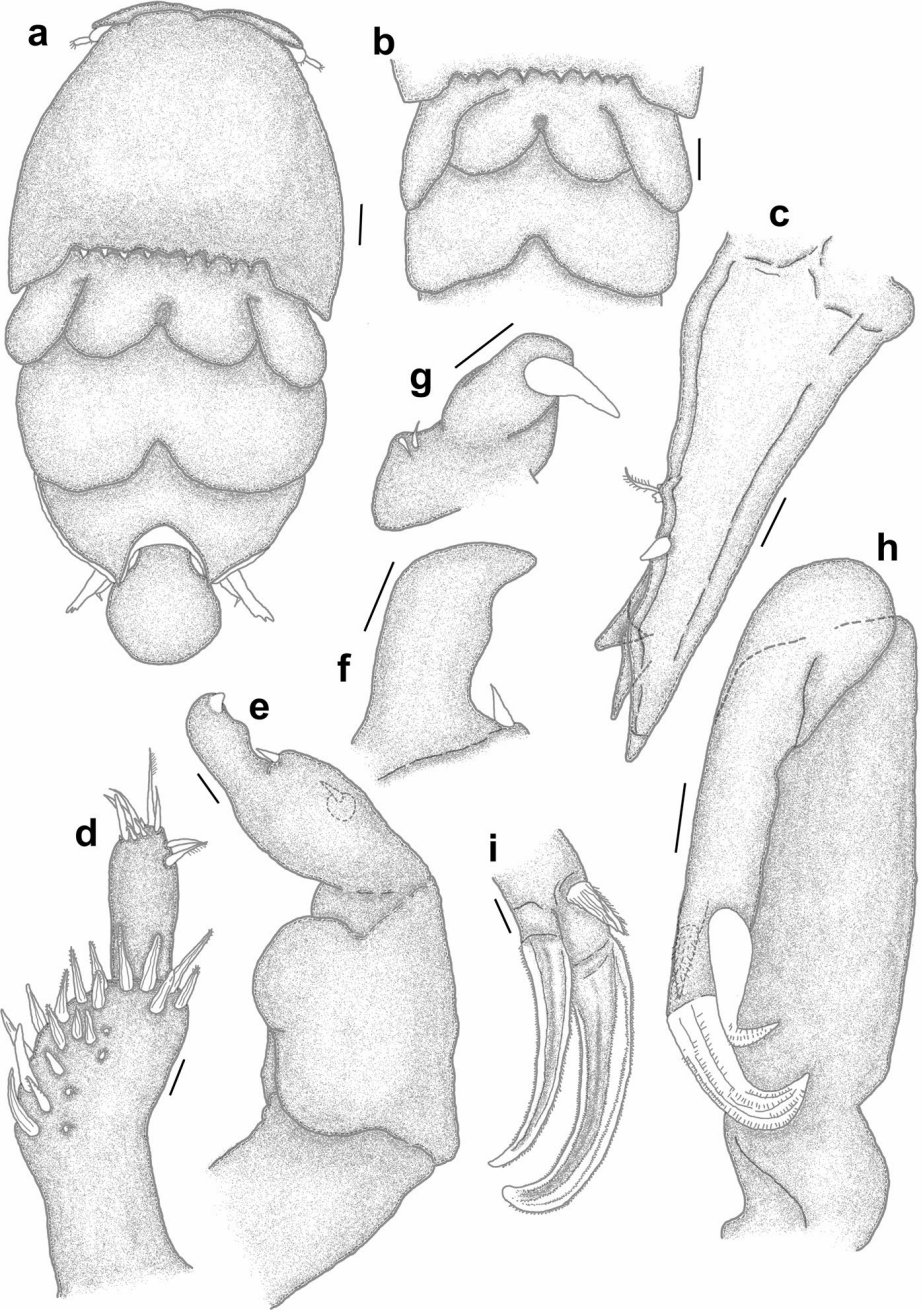
*Pandarus sinuatus* Say, 1818. a, adult female; b, dorsal thoracic plates of another specimen; c, caudal ramus; d, antennule; e, antenna; f, terminal claw of antenna; g, maxillule; h, maxilla; i, distal part of maxilla with calamus, canna and clavus. Scale-bars: a, b, 0.5 mm; c, h, 100 µm; d, e, f, g, i, 50 µm.

**Fig. 8:**
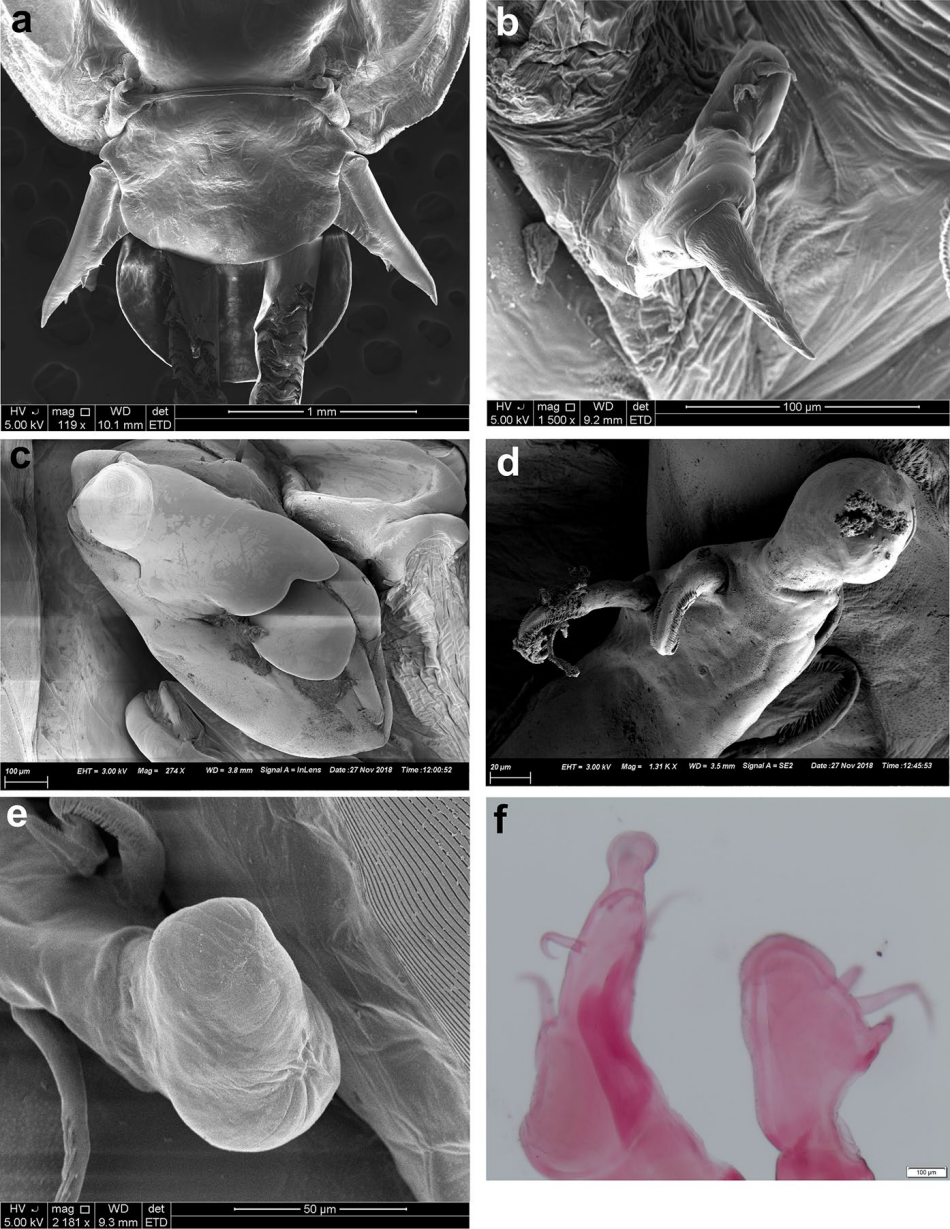
*Pandarus sinuatus* Say, 1818. a, scanning electron micrograph of abdomen and caudal rami, ventral view; b, scanning electron micrograph of maxillule; c, scanning electron micrograph of maxilliped; d, scanning electron micrograph of bulbous distomedial process of leg 1 exopod distal segment; e, scanning electron micrograph of distomedial bulbous process of leg 1 exopod distal segment (different view); f, light microscope photo of leg 1 exopod with distomedial bulbous process of distal segment and distal segment of endopod.

*Pandarus* Leach, 1816 is one of the 23 genera of the family Pandaridae Milne Edwards, 1840 (Walter & Boxshall 2024) mostly infecting elasmobranchs, with *Pandarus* specimens commonly found on the body surface of the host (Cressey 1967; Kabata 1979). Currently there are 14 accepted species of *Pandarus* (Walter & Boxshall 2024), namely *P*. *bicolor* Leach, 1816; *P*. *sinuatus* Say, 1818; *P*. *cranchii* Leach, 1819; *P*. *rouxii* Risso, 1826; *P*. *satyrus* Dana, 1849; *P*. *zygaenae* Brady, 1883; *P*. *brevicaudis* Dana, 1852–1853; *P*. *smithii* Rathbun, 1886; *P*. *ambiguus* (Scott T., 1907); *P*. *niger* Kirtisinghe, 1950; *P*. *carcharhini* Ho, 1963; *P*. *floridanus* Cressey, 1967; *P*. *katoi* Cressey, 1967, and *P*. *rhincodonicus* Norman, Newbound & Knott, 2000. However, in the revision of the pandarids, Cressey (1967) made no mention of *P*. *rouxii*, *P*. *brevicaudis* or *P*. *ambiguus*. Carus (1885) referred to *P*. *rouxii* as a “*species non determinanda*” and thus should be a *species inquirenda*. Additionally, *P*. *brevicaudis* was accepted by Wilson (1907) as a valid species and described the female and male based on specimens described by Dana (1853) as *P*. *brevicaudis* and *Nogagus validus*, respectively. However, comparing Dana’s (1853) illustrations (see Figs. 2a and 3a on plate 95) of the females of *P*. *satyrus* and *P*. *brevicaudis* respectively, it is obvious that Fig. 3a represents an immature female. Wilson (1907) also mentioned *P*. *brevicaudatus* from Bassett-Smith (1899) as a synonym of *P*. *brevicaudis* which Bassett-Smith (1899) referred to as “imperfectly described”. Thus, *P*. *brevicaudis* should also be regarded as a *species inquirenda*. Regarding *P*. *ambiguus* described as *Nogagus ambiguus* in Scott & Scott (1913), the description and illustrations (see Figs. 1–8 of plate XX) is that of an immature pandarid (*cf*. Fig. 5A in Izawa (2010)), most likely a male due to the claw on the maxilliped (see Fig. 4). Comparing this description and illustration with those of the copepodid stages of male and female *P*. *cranchii* (Izawa 2010) discrepancies are observed especially regarding the structure of leg 4 of *N*. *ambiguus* (i.e. *P*. *ambiguus*) (see Fig. 8 of plate XX in Scott & Scott (1913)) with a 1-segmented exopod and 2-segmented endopod while leg 4 of the copepodid stages and adult female of *P*. *cranchii* have 1-segmented rami and the adult male has 2-segmented rami, similar to the adults of other *Pandarus* species (Cressey 1967). Therefore, *P*. *ambiguus* should also be regarded as a *species inquirenda* and consequently there are currently only 11 valid species.

According to Cressey (1967), the adult females can be divided into two groups based on their dorsal morphology, i.e., the “bicolor” group with the dorsal plates of thoracic somite two that extends only up to the posterior edge of the plate of somite three (including *P*. *bicolor*, *P*. *niger*, *P*. *carcharini*) and the “cranchii” group with the dorsal plates of the thoracic somite two extending well beyond the posterior edge of that of somite three (including *P*. *sinuatus*, *P*. *cranchii*, *P*. *satyrus*, *P*. *zygaenae*, *P*. *smithii*, *P*. *floridanus*, *P*. *katoi* and *P*. *rhincodonicus*). The three species in the “bicolor” group can be distinguished from each other mainly by the lengths of the caudal rami with those of *P*. *bicolor* barely visible in dorsal view, those of *P*. *carcharhini* just extending beyond the abdominal plate and those of *P*. *niger* extending well beyond the abdominal plate (Kirtisinghe 1950; Ho 1963; Cressey 1967). In the “cranchii” group, *P*. *satyrus*, *P*. *cranchii* and *P*. *rhincodonicus* are morphologically very similar (Cressey 1967; Norman et al. 2000) as are *P*. *floridanus* and *P*. *sinuatus*; and *P*. *katoi* and *P*. *zygaenae* (Cressey 1967) while *P*. *smithii* can be distinguished from all the other species in the “cranchii” group by the presence of basal medial expansions on the caudal rami (Izawa 2010). However, *Pandarus* species undergo considerable morphological changes during their ontogeny (Kabata 1979) as well as varying signs of pigmentation (Ho 1963; Cressey 1967; Kabata 1979) and therefore using their dorsal features for species identification may result in misidentifications. Additionally, the setation of the limbs also exhibits variability (Hewitt 1967) with some species having the same spine and setal formulas (Cressey 1967).

Current reports of *Pandarus* species from South African waters include *P*. *bicolor* from *Carcharias* sp., dogfish, *Galeorhinus galeus* (Linnaeus), *Odontaspis* sp., *Squalus acanthias* (Linnaeus) (Dippenaar 2004) and *Carcharodon carcharias* (Linnaeus), *Mustelus palumbes* Smith, *Notorynchus cepedianus* (Péron), and *Triakis megalopterus* (Smith) (Dippenaar 2024); *P*. *carcharini* from *Carcharhinus leucas* (Valenciennes) (Dippenaar 2004); *P*. *niger* from *Carcharhinus obscurus* (LeSueur) (Dippenaar 2024); *P*. *cranchii* from *Carcharhinus longimanus* (Poey), *Carcharodon carcharias*, *Poroderma africanum* (Gmelin), *Sphyrna zygaena* (Linnaeus), *Stegostoma tigrinum* (Foster), (Dippenaar 2004), *Isurus oxyrinchus* (Rafinesque) and *Sphyrna lewini* (Griffith & Smith) (Dippenaar 2024); *P*. *floridanus* from *Carcharias taurus* Rafinesque (Dippenaar 2004); and *P*. *smithii* from *Carcharhinus* sp., *C*. *obscurus*, *Carcharias* sp., *C*. *taurus*, *Carcharodon carcharias*, *I*. *oxyrinchus*, *Odontaspis* sp., *Prionace glauca* (Linnaeus), *Rhincodon typus* Smith, *Rhizoprionodon acutus* (Rüppell), (Dippenaar 2004), *Carcharhinus brachyurus* (Günther), *C*. *limbatus*, *Galeocerdo cuvier* (Péron & LeSueur), *S*. *lewini* (Dippenaar 2024).

In the present study, species of *Pandarus* were collected from elasmobranchs caught from both the Atlantic and Indian oceans off South Africa. Thus, the paper reports on eight *Pandarus* species collected with additional notes on their characteristic features and redescribes and illustrates the adult females of *P*. *satyrus* and *P*. *sinuatus*, including new hosts and/or geographical records of some collected species. Additionally, a new species, *P*. *echinifer* **n. sp.**, is described. Sequences divergences of 13 individuals (10 downloaded from Genbank and three newly generated) using the cytochrome oxidase I partial gene were used to estimate inter- and intraspecific divergences within and amongst *Pandarus* species.

## Materials and Methods

### Sampling and morphological observation

Copepod specimens were collected from elasmobranch species caught mostly in the nets of the KwaZulu-Natal Sharks Board (KZNSB) and some species caught as by-catch during hake assessment demersal cruises off the west coast of South Africa on board the Department of Agriculture, Forestry and Fisheries (DAFF) research vessel *R/V* (*Africana*) during 2008 as well as from fish caught by commercial fishermen off Gansbaai (West coast). Additionally, specimens were collected from white sharks during the Ocearch Project in South Africa as well as from a snaggletooth shark from uShaka Marine World, Durban. Furthermore, specimens were collected from a stranded whale shark off the west coast while collected specimens from whale sharks swimming in Ningaloo Marine Park (Western Australia) were also obtained. The fish hosts were mostly identified by scientists at KZNSB and researchers on board the vessels. Collected specimens were fixed and preserved in 70% ethanol. Selected specimens were cleared and stained in lactic acid with a small amount of dissolved lignin pink. These specimens were dissected and studied under both stereo- and light microscopes using the wooden slide technique (Humes & Gooding 1964). Selected specimens were prepared for scanning electron microscopy (SEM) by dehydrating them through a series of ethanol (70, 80, 90, 100, 100% for about 30 min each) followed by immersion in hexamethyldisilazane for about an hour. Excess hexamethyldisilizane, not evaporated, were removed and the specimens were allowed to dry completely before being sputter-coated with gold-palladium and carbon. Host names were verified using Froese & Pauly (2024). Morphological nomenclature mostly follows Cressey (1967) and Kabata (1979). Voucher specimens and the type-material were deposited in the Iziko South African Museum, Cape Town, South Africa.

### DNA extraction and sequencing

Genomic DNA was extracted from specimens of *Pandarus* collected from the stranded whale shark in South Africa as well as from specimens received from Ningaloo Marine Park (Western Australia) using the QIAGEN® QIAamp® DNA micro kit (Whitehead Scientific (Pty) Ltd) according to the manufacturer’s instructions. Polymerase Chain Reaction (PCR) was used to amplify a fragment of the mitochondrial (mtDNA) COI (Cytochrome Oxidase I) gene using primers LCO 1490 (forward) and HCO 2198 (reverse) (Folmer et al. 1994). The 20 µl PCR reaction mixture consisted of 10 µl Master mix (Ampliqon Taq DNA Polymerase Master Mix RED) containing HotStarTaq Plus DNA polymerase, 1 µl of each primer, 5–8 µl (depending on the DNA concentration) of DNA and ddH_2_O to top up the volume. The cycling conditions consisted of an initial denaturation at 95 °C (4 min); followed by 30 cycles of 94 °C (1 min) denaturation, 43–45 °C (2 min) annealing and 72 °C (3 min) extension, with a final extension of 72 °C (10 min) in the MiniOpticon real-time PCR system. Purification of PCR products and sequencing was done by Inqaba Biotechnical Industries (Pty) Ltd. The resulting chromatograms of the sequences were checked for nucleotide ambiguities, and the forward and reverse sequences assembled and edited using CLC main workbench 7.9.1 (QIAGEN). Generated sequences were aligned with sequences downloaded from Genbank (Accession numbers: HG942363, FJ447387–FJ447391, KJ551843 and OL457303–OL457305) using Clustal X 2.0.12 (Thompson et al. 1997). The aligned dataset was imported into MacClade 4.0 (Maddison & Maddison 2001) and translated into amino acids to verify the alignment. Uncorrected pairwise sequence divergences were estimated using MEGA 7 (Kumar et al. 2016). The generated sequences were submitted to Genbank (Accession numbers: PP434798–PP434800).

## Results

### Systematics

**Family Pandaridae Milne Edwards, 1840**

**Genus** *Pandarus* Leach, 1816

***Pandarus bicolor*** **Leach, 1816**

*Hosts: Mustelus palumbes* Smith; *M*. *mustelus* (Linnaeus); *Galeorhinus galeus* (Linnaeus); *Triakis megalopterus* (Smith) (Carcharhiniformes: Triakidae); *Notorynchus cepedianus* (Péron) (Hexanchiformes: Hexanchidae), and *Carcharodon carcharias* (Linnaeus) (Lamniformes: Lamnidae) from west coast (SA).

*Locality:* Atlantic Ocean, South Africa.

*Material examined:* Several ♀♀ from all different host species.

*Voucher material:* Two adult ♀♀ (SAMC-A096821) collected from *M*. *mustelus*; four adult ♀♀ (SAMC-A096822) collected from *G. galeus;* two adult ♀♀ (SAMC-A096823) collected from *N. cepedianus;* two adult ♀♀ (SAMC-A096824) collected from *C*. *carcharias*.

*Pandarus bicolor* females can be easily identified from all other species by a combination of the relative lengths of the dorsal thoracic plates and the structure of the caudal rami. The dorsal plates (Fig. 1a) of the second thoracic somite extend only to the posterior margin of those of the third thoracic somite (see Fig. 2 in Öktener et al. (2020) and Fig 769 in Kabata (1979)) while the caudal rami (Fig. 1b) are short and broad, barely visible in dorsal view (Fig. 1a) (see Fig. 3 in Öktener et al. (2020) and Fig 771 in Kabata (1979)).

*Pandarus niger* Kirtisinghe, 1950

*Hosts: Carcharhinus obscurus* (Lesueur) (Carcharhiniformes: Carcharhinidae) from east coast (SA).

*Locality:* Indian Ocean, South Africa.

*Material examined:* 1♀ from single host specimen.

*Pandarus niger* females are easily identified from all other species by the same combination of features as *P*. *bicolor*, namely the relative lengths of the dorsal thoracic plates and the appearance of the caudal rami. The dorsal plates of the second thoracic somite extend only to the posterior margin of those of the third thoracic somite (Fig. 1c) while the caudal rami are long and reach well beyond the posterior margin of the dorsal abdominal plate (Fig. 1c) (caudal rami about 1^1^/_3_ times as long as the dorsal abdominal plate (Rangnekar & Rangnekar 1972) (see Plate 1 in Rangnekar & Rangnekar (1972)).

*Pandarus carcharhini* Ho, 1963

*Hosts: Carcharhinus leucas* (Valenciennes) (Carcharhiniformes: Carcharhinidae) from east coast (SA).

*Locality:* Indian Ocean, South Africa.

*Material examined:* 1♀ from single host specimen.

*Pandarus carcharhini* females are distinguished from all other species by the same combination of features as *P*. *bicolor* and *P*. *niger*, namely that the dorsal plates of the second thoracic somite extend only to the posterior margin of those of the third thoracic somite (Fig. 1d) while the caudal rami are longer than those in *P*. *bicolor* reaching beyond the posterior margin of the dorsal abdominal plate (Ho 1963) (although slightly shorter than abdominal plate in current specimen), but shorter than those of *P*. *niger* (see Fig. 13 in Ho (1963)).

*Pandarus cranchii* Leach, 1819

*Hosts: Sphyrna lewini* (Griffith & Smith) (Carcharhiniformes: Sphyrnidae); *Carcharodon carcharias* (Linnaeus); *Isurus oxyrinchus* Rafinesque (Lamniformes: Lamnidae) from east coast (SA), and *Rhincodon typus* Smith (Orectolobiformes: Rhincodontidae) from Ningaloo Marine Park (Western Australia).

*Locality:* Indian Ocean, South Africa and Ningaloo Marine Park (Western Australia).

*Material examined:* Several ♀♀ from all host specimens.

*Voucher material:* Four adult ♀♀ (SAMC-A096825) collected from *S. lewini*; one adult ♀ (SAMC-A096826) collected from *C. carcharias;* two adult ♀♀ (SAMC-A096827) collected from *I. oxyrinchus;* one adult ♀ (SAMC-A096828) collected from *R*. *typus* from Western Australia.

*Pandarus cranchii* females have dorsal plates of the second thoracic somite that extend well beyond the posterior margin of those of the third thoracic somite (Figs. 2a, b), sometimes even beyond the posterior margin of the plate of the fourth thoracic somite (Fig. 2a). The caudal rami extend mostly beyond the posterior margin of the dorsal abdominal plate (Fig. 2a) or at least up to the posterior margin (see Fig. 2b and Fig. 1A in Izawa (2010)). Additionally, the second segment of leg 1 exopod is armed with a distomedial spinulated process (Figs. 2c, d) (see Fig. 2A in Izawa (2010)).

*Pandarus satyrus* Dana, 1849

*Hosts: Carcharodon carcharias* (Linnaeus); *Isurus oxyrinchus* Rafinesque (Lamniformes: Lamnidae); *Carcharias taurus* Rafinesque (Lamniformes: Carchariidae) from east coast (SA), and *Rhincodon typus* Smith (Orectolobiformes: Rhincodontidae) from west coast (SA) and Ningaloo Marine Park (Western Austalia).

*Locality:* Indian and Atlantic Oceans, South Africa and Ningaloo Marine Park (Western Australia).

*Material examined:* Several ♀♀ from all host specimens.

*Voucher material:* Five adult ♀♀ (SAMC-A096829) collected from *I. oxyrinchus*; five adult ♀♀ (SAMC-A096830) collected from *R*. *typus*.

### Re-description (Figs. 3, 4)

*Adult female* [based on 10 specimens]. Cephalothorax dorsally with several small denticles medially on posterior margin (Fig. 3a). Dorsal thoracic plates of second somite about twice as long as that of third somite reaching more than half length of dorsal plate of fourth somite (sometimes almost to posterior margin of dorsal plate of fourth somite (Fig. 3b)). Genital complex with paired posterior protrusions each armed with a small spine (Figs. 3c, d). Dorsal abdominal plate fan-shaped with small spinule on lateral margin (Fig. 3c). Caudal rami (Fig. 3e) lateral to dorsal abdominal plate, extending beyond middle of dorsal abdominal plate (Fig. 3a) (sometimes beyond posterior margin of dorsal abdominal plate (Fig. 3b)), sharply pointed distally with 2 processes and pinnate seta dorsally and 1 spine and small pinnate seta ventrally.

Antennule (Fig. 3f), antenna (Figs. 3g, 4a), oral cone (Fig. 4b), mandible (Figs. 3h, 4b), maxillule (Figs. 3i, 4c), maxilla (Figs. 3j, 4d) and maxilliped (Fig. 4e) mostly similar to those of *P*. *cranchii* with small differences in the number of setae on the antennule and a possible additional small seta on the palp of the maxillule (not observed in all examined specimens).

Legs 1–3 biramous, 2-segmented, leg 4 1-segmented. Armature formula as follows with spines (Roman numerals) and setae (Arabic numerals):

**Table:** 

	Endopod		Exopod	
	1	2	1	2
Leg 1	0-0	3	I-0	III, I, 3
Leg 2	0-0	4	I-0	III, I, 6
Leg 3	0-0	2	I-0	III, I, 2
Leg 4	0(1)	-	IV, I, 1	

Distomedial spine on leg 1 exopod bifid, shoe-shaped (Fig. 4f). Leg 5 (Fig. 3k) with inner conical process armed with small spine and outer small pinnate seta.

### Remarks

Females of *P*. *satyrus* and *P*. *cranchii* are morphologically very similar and can apparently be distinguished by the lengths of the caudal rami relative to the length of the dorsal abdominal plate (Cressey 1967; Lewis 1966), while the leg armature is mostly the same for the two species (Cressey 1967). However, even though the plates of the second thoracic somite of *P*. *cranchii* mostly extends to or beyond the posterior margin of the plate of the fourth thoracic somite some individuals of *P*. *satyrus* have equally long plates of the second thoracic somite (current observations, see Fig. 3b). Furthermore, the caudal rami of *P*. *satyrus* mostly do not extend beyond the posterior margin of the dorsal abdominal plate, as often seen in *P*. *cranchii* (with exceptions, see Fig. 12D in Shiino (1954)), but some individuals also have equally long caudal rami as seen in *P*. *cranchii* (current observations, see Fig. 3b). Due to the variation in the armature of the legs present in members of Pandaridae (Hewitt 1967) reports of different numbers of armature elements are not unusual for *P*. *satyrus* females (e.g. Shiino 1954; Ho 1963; Lewis 1966; Cressey 1967; Hewitt 1967). However, a clear difference between *P*. *satyrus* and *P*. *cranchii* females is the structure of the distomedial spine on the last exopodal segment of leg 1 with that of *P*. *cranchii* being a spinulated process (see Figs. 2c, d and Fig. 2A in Izawa (2010), Fig. 13B in Shiino (1954), Fig. 12c in Lewis (1966) and Fig. 145 in Hewitt (1967)) while that of *P*. *satyrus* is a smooth, bifid process (see Fig. 4f and Fig. 9b in Lewis (1966)).

*Pandarus smithii* Rathbun, 1886

*Hosts: Carcharhinus brachyurus* (Günther) from west coast (SA); *C*. *limbatus* (Valeniennes); *C*. *brevipinna* (Valenciennes); *C*. *obscurus* (LeSueur) (Carcharhiniformes: Carcharhinidae); *Galeocerdo cuvier* (Péron & LeSueur) (Carcharhiniformes: Galeocerdonidae); *Sphyrna lewini* (Griffith & Smith) (Carcharhiniformes: Sphyrnidae) from east coast (SA); *Carcharodon carcharias* (Linnaeus) from east and west coasts (SA); *Isurus oxyrinchus* Rafinesque (Lamniformes: Lamnidae) from east coast (SA), and *Rhincodon typus* Smith (Orectolobiformes: Rhincodontidae) from west coast (SA).

*Locality:* Indian and Atlantic Oceans, South Africa.

*Material examined:* Several ♀♀ from all host specimens.

*Voucher material:* Six adult ♀♀ (SAMC-A096831) collected from *C. carcharias*; four adult ♀♀ (SAMC-A096832) collected from *I*. *oxyrinchus*.

*Pandarus smithii* females have dorsal plates of the second thoracic somite that extend beyond the posterior margin of those of the third thoracic somite, almost reaching middle of plate on fourth thoracic somite (Fig. 5a, see Fig. 34 in Cressey (1967) and Fig. 7A in Izawa (2010)). The caudal rami are mostly shorter than the dorsal abdominal plate or extend almost to the posterior margin of the dorsal abdominal plate (Fig. 5a, see Fig. 34 in Cressey (1967) and Fig. 7A in Izawa (2010)) bearing basal medial expansions (often shorter than posterior margin of abdomen (see Figs. 5b, 6a). The second segment of leg 1 exopod is armed with a distomedial smooth process with a slightly extended tip (Fig. 6b, c, d) (sometimes causing a slightly bifid appearance (Figs. 6c, d) (see Fig. 8A in Izawa (2010)).

*Pandarus sinuatus* Say, 1818

*Hosts: Carcharias taurus* Rafinesque (Lamniformes: Carchariidae); *Carcharhinus leucas* (Valenciennes); *C*. *plumbeus* (Nardo) (Carcharhiniformes: Carcharhinidae); *Sphyrna mokarran* (Rüppell) (Carcharhiniformes: Sphyrnidae).

*Locality:* Indian Ocean, South Africa.

*Material examined:* Several ♀♀ from all host specimens.

*Voucher material:* 10 adult ♀♀ (SAMC-A096833) collected from *C. taurus*.

### Re-description (Figs. 7, 8, 9)

*Adult female* [based on 10 specimens]. Body typical for *Pandarus* morphology. Cephalothorax dorsally with serrated posteromedial margin with about 4 sharp denticles on either side of middle (Fig. 7a). Dorsal thoracic plates of second somite slightly longer than that of third somite (sometimes appearing almost equal in length), reaching only about a third length of dorsal plate of fourth somite (Fig. 7a) (some specimens reaching about half length of dorsal plate of fourth somite (Fig. 7b)). Genital complex with paired posterior protrusions (Fig. 7a). Dorsal abdominal plate mostly circular with thin anterior extension joining with genital complex (Fig. 7a). Caudal rami (Figs. 7a, 8a) lateral to dorsal abdominal plate, extending slightly beyond middle of dorsal abdominal plate (Fig. 7a), slender, sharply pointed distally with 2 processes dorsally and 1 spine and small pinnate seta ventrally (Fig. 7c), slight basal medial expansions (Figs. 7a, 8a).

Antennule (Fig. 7d) 2-segmented with 22 and 11 setae on first and second segments, respectively. Antenna (Fig. 7e) similar to other *Pandarus* females with slightly reduced terminal claw (Fig. 7f). Oral cone and mandible similar to congeneric members. Maxillule (Figs. 7g, 8b) endite with stout distal process, palp with 2 (sometimes 3) small setae. Maxilla (Fig. 7h) brachiform, brachium with calamus, cana and clavus (Fig. 7i). Maxilliped (Fig. 8c) similar to congeneric members.

**Fig. 9:**
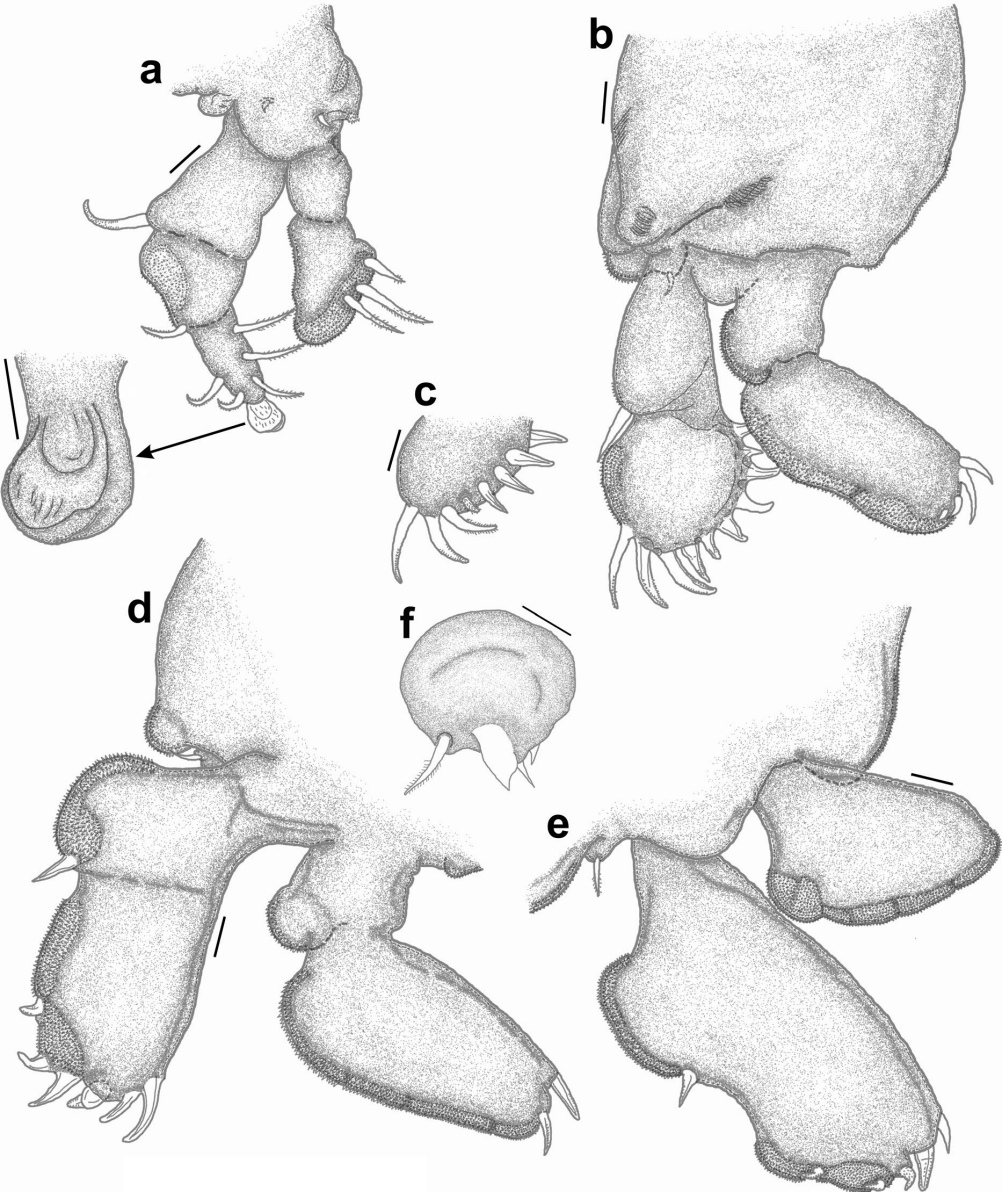
*Pandarus sinuatus* Say, 1818. a, leg 1 with enlarged distomedial process (50 µm) of last exopod segment; b, leg 2; c, distal part of last exopod segment leg 2 (different view); d, leg 3; e, leg 4; f, leg 5. Scale-bars: a, b, c, d, e, 100 µm; f, 50 µm.

Legs 1–3 (Figs. 9a–d), biramous, 2-segmented, leg 4 (Fig. 9e) 1-segmented. Armature formula as follows with spines (Roman numerals) and setae (Arabic numerals):

**Table:** 

	Endopod		Exopod	
	1	2	1	2
Leg 1	0-0	3	I-0	III, I, 3
Leg 2	0-0	4	I-0	III, I, 6
Leg 3	0-0	2	I-0	III, I, 2
Leg 4	0	-	IV, I, 1	

Distomedial spine on leg 1 exopod smooth, bulbous ((Figs. 8d–f, 9a). Leg 5 (Fig. 9f) small, pointed tubercle and two small setae with adjacent pinnate seta.

### Remarks

Females of *P*. *sinuatus* are part of the “cranchii group” with the dorsal plates of thoracic somite two extending “well beyond” the posterior margin of the plate of somite three (Cressey 1967) even though the plates of somite 2 only extend slightly beyond the plate of somite three. Furthermore, *P*. *sinuatus* is related to *P*. *floridanus* but are distinguishable by the lengths of the plates of thoracic somite 2, with those of *P*. *floridanus* extending beyond the middle of the plate of somite 4 whereas those of *P*. *sinuatus* are not extending to the middle of the plate of somite 4, but only to the anterior third (Cressey 1967). Additionally, the dorsal abdominal plate is about as long as the exposed caudal rami (Cressey 1967). However, current observations highlight the fact that the plates of thoracic somites two and three are often almost the same length (see Fig. 7a) while these plates may sometimes extend further than just the anterior third of the plate of somite four (see Fig. 7b). Additionally, the length of the caudal rami of the studied specimens were mostly shorter than the length of the dorsal abdominal plate (see Fig. 7a) rather than the same length (according to Wilson (1907)). The presence of slight basal medial expansions on the caudal rami of *P*. *sinuatus* individuals (see Figs. 7a, 8a) as well as the variation in the sizes and shapes of the dorsal thoracic plates resulted in difficulty distinguishing between females of *P*. *sinuatus* and *P*. *smithii*. However, the structure of the distomedial spine on the last exopodal segment of leg 1 can be used to distinguish the females of the two species, with that of *P*. *sinuatus* appearing much more bulbous (Figs. 8d–f) and not slightly bifid (Figs. 6b–d) like that of *P*. *smithii*.

*Pandarus echinifer* **n. sp.**

*Type-host: Hemipristis elongata* (Klunzinger) (Carcharhiniformes: Hemigaleidae).

*Type-locality:* Indian Ocean, South Africa.

*Material examined:* Two adult ♀♀ from a single host specimens collected on 23rd of February 2005 from uShaka Marine World, Durban.

*Type-material:* One adult ♀ (holotype) (SAMC-A096834). Remaining female (1 dissected) retained in the personal collection of the author.

*ZooBank registration:* To comply with the regulations set out in Article 8.5 of the amended 2012 version of the *International Code of Zoological Nomenclature* (ICZN, 2012), details of the new species have been submitted to ZooBank. The Live Science Identifier (LSID) for *Pandarus echinifer* n. sp. is urn:lsid:zoobank.org:pub:B4AB53D1-D770-4BAF-8992-51D3646B8F13.

*Etymology*: The specific name *echinifer* (sea-urchin bearer) refers to the structure of the distomedial spine on the last exopodal segment of leg 1 that reminds of the spines on a sea-urchin.

### Description (Figs. 10, 11, 12)

**Fig. 10:**
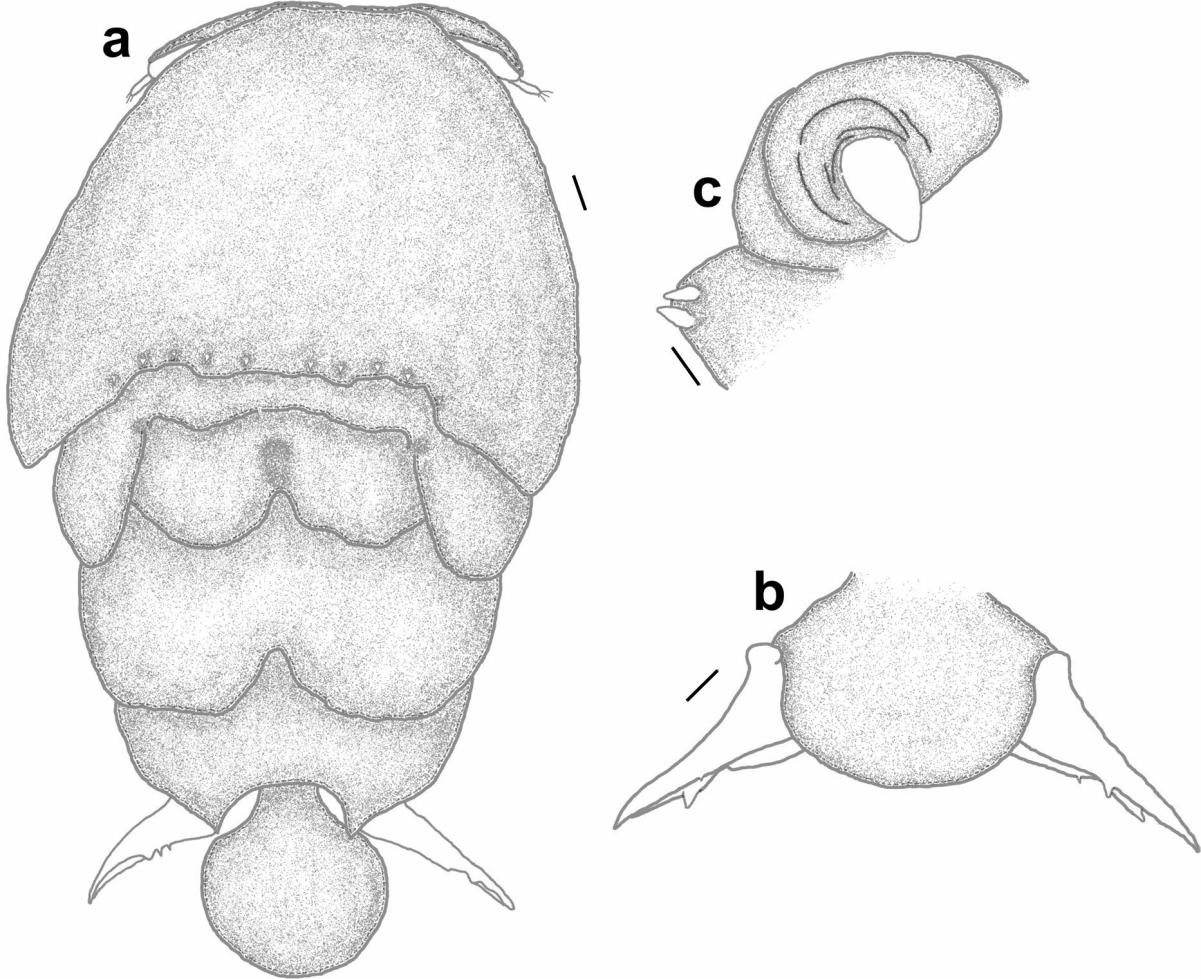
*Pandarus echinifer* **n. sp.** a, adult female, b, abdomen and caudal rami, ventral view; c, maxillule. Scale-bars: a, 2.5 mm; b, 0.2 mm, c, 10 µm.

*Adult female* [Based on two specimens, one without abdominal plate]. Female typical *Pandarus* morphology. Body length from anterior margin to posterior margin of abdominal plate/abdomen 6.25–6.5 mm (mean 6.4), cephalothorax length 2.5–2.8 mm (mean 2.65), cephalothorax width 3.75–3.85 mm (mean 3.8), abdominal plate length and width 1.25 mm. Cephalothorax dorsally with uneven posteromedial margin with several sharp denticles (about 5 on either side of middle) (Fig. 10a). Dorsal thoracic plates of second somite slightly longer than that of third somite, reaching only about a third length of dorsal plate of fourth somite (Fig. 10a). Genital complex with paired posterior protrusions (Fig. 10a). Dorsal abdominal plate mostly circular with narrow anterior extension joining with genital complex (Fig. 10a). Caudal rami (Fig. 10a) lateral to dorsal abdominal plate, extending beyond middle of dorsal abdominal plate, slender, sharply pointed distally with 2 processes dorsally and 1 spine and small pinnate seta ventrally, slight basal medial expansions (Fig. 10b).

Antennule, antenna, oral cone, mandible, maxillule (Fig. 10c), maxilla and maxilliped mostly similar to congeneric members.

**Fig. 11:**
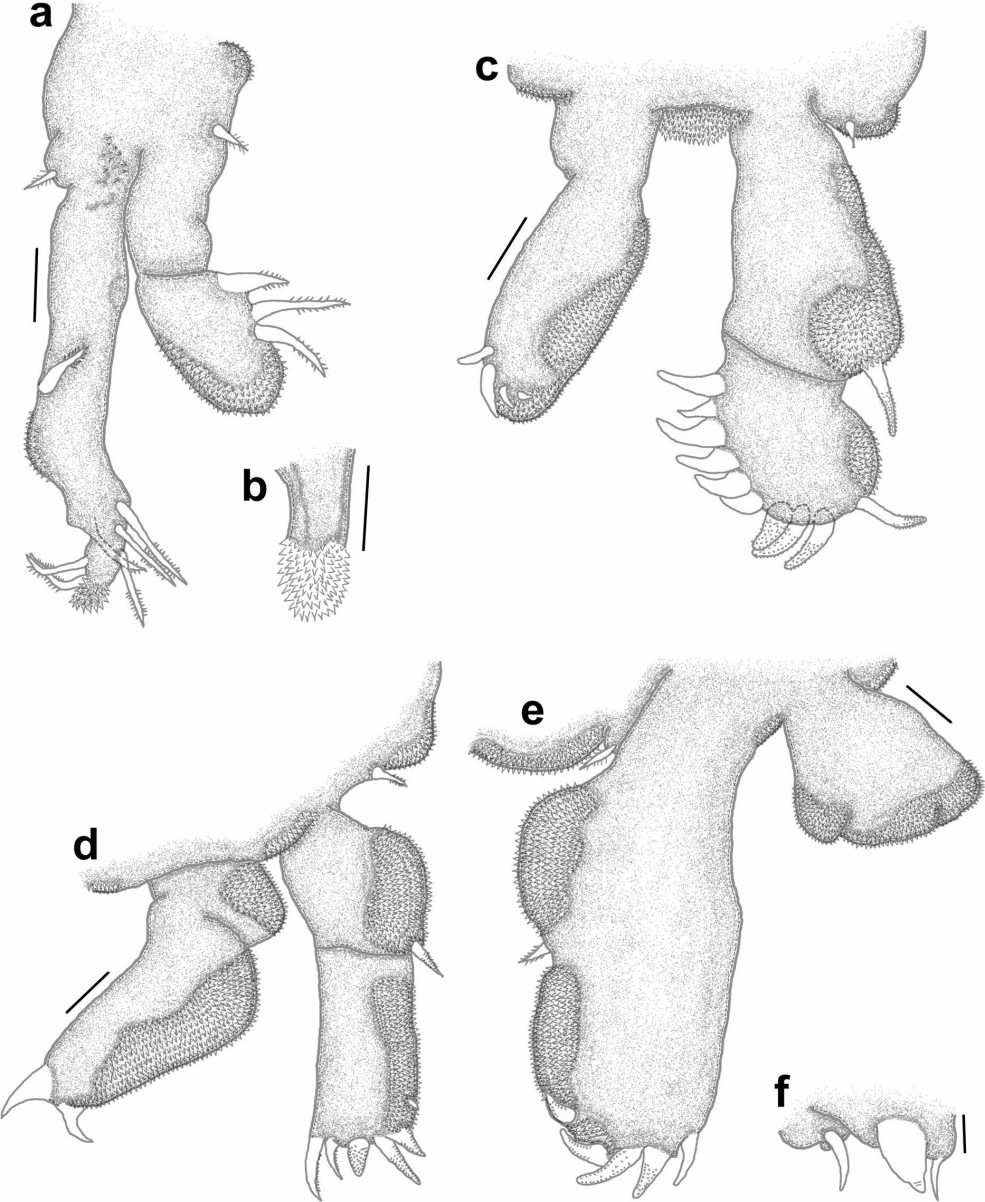
*Pandarus echinifer* **n. sp.** a, leg 1; b, distomedial spine of last exopod segment leg 1; c, leg 2; d, leg 3; e, leg 4; f, leg 5. Scale-bars: a, c, d, e, 100 µm; b, f, 50 µm.

Legs 1–3 (Figs. 11a–d), biramous, 2-segmented, leg 4 (Fig. 11e) 1-segmented. Armature formula as follows with spines (Roman numerals) and setae (Arabic numerals):

**Table:** 

	Endopod		Exopod	
	1	2	1	2
Leg 1	0-0	3	I-0	III, I, 3
Leg 2	0-0	4	I-0	III, I, 6
Leg 3	0-0	2	I-0	III, I, 2
Leg 4	0	-	IV, I, 1	-

**Fig. 12:**
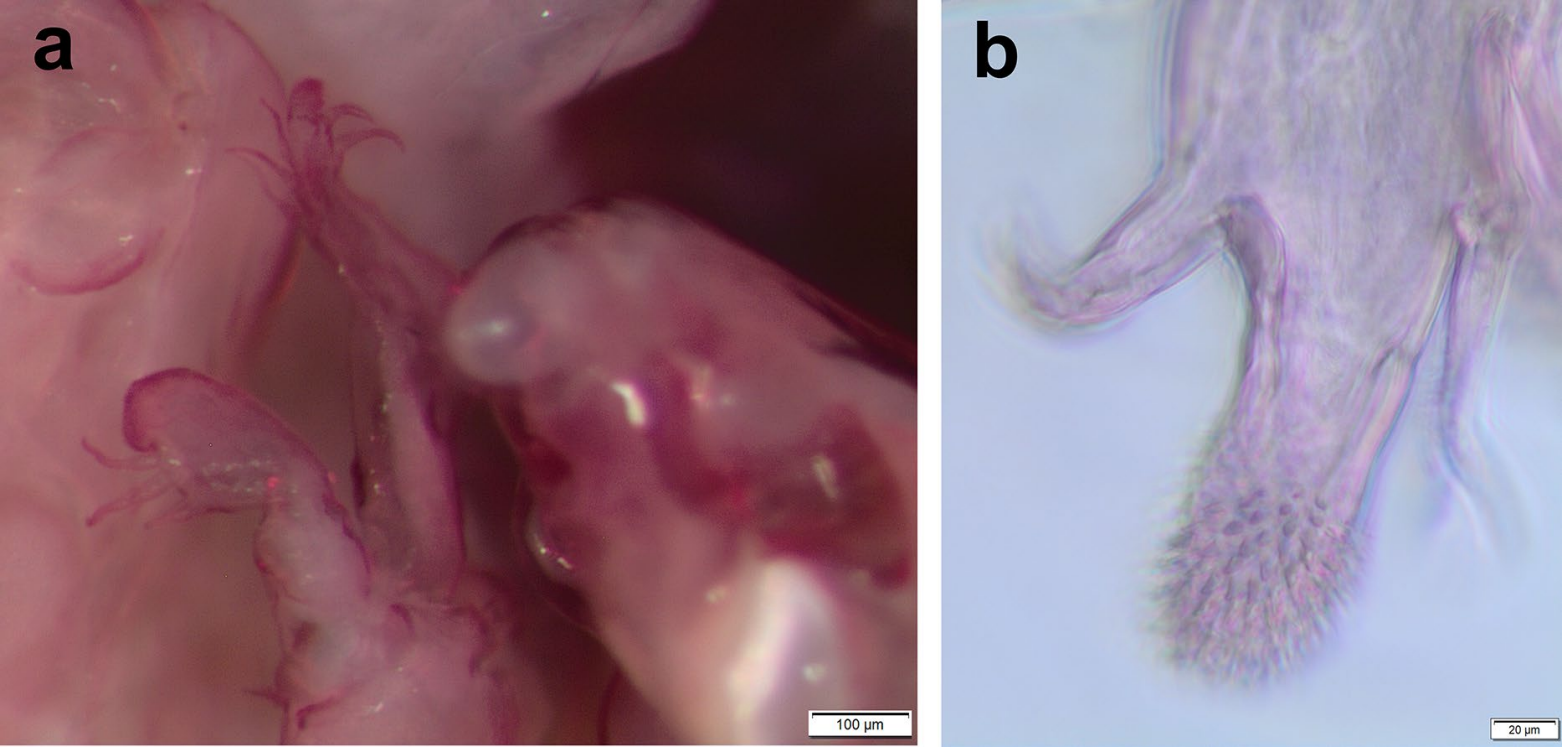
*Pandarus echinifer* **n. sp.**, light microscope photos. a, leg 1; b, distal part of last exopod segment leg 2.

Distomedial spine on leg 1 exopod heavily spinulated ((Figs. 11b, 12a, b). Leg 5 (Fig. 11f) small pointed tubercle and one small seta with adjacent seta.

### Remarks

Females of *P*. *echinifer ***n. sp.** are also part of the “cranchii group” with the dorsal plates of thoracic somite two extending “well beyond” the posterior margin of the plate of somite three (Cressey 1967), although they are only slightly longer than the plate of somite three. Additionally, the plates of thoracic somite two extends only to the anterior third of the plate of somite four while the dorsal abdominal plate is longer than the exposed caudal rami. These features are very similar to those *P*. *sinuatus*. Additionally, the presence of slight basal medial expansions on the caudal rami of *P*. *echinifer ***n. sp.** individuals similar to those in *P*. *sinuatus* also cause difficulty distinguishing among females of *P*. *echinifer ***n. sp.**, *P*. *sinuatus* and *P*. *smithii*. The structure of the distomedial spine on the last exopodal segment of leg 1 can be used to distinguish among the three species with similar dorsal morphology i.e. *P*. *echinifer ***n. sp.**, *P*. *sinuatus* and *P*. *smithii* with that of *P*. *echinifer ***n. sp.** heavily spinulated (Figs. 11b, 12a, b), that of *P*. *sinuatus* appearing more bulbous (Figs. 8d–f, 9a) and that of *P*. *smithii* a bulbous process with a slightly extended tip, giving it a slightly bifid appearance (Figs. 6b–d).The spinules covering the tip of the distomedial spine on the last exopodal segment of leg 1 of *P*. *echinifer ***n. sp.** is reminiscent of that of *P*. *cranchii* (see Fig. 2c, d) but with more prominent spinules (see Fig. 12b) while the relative lengths of the dorsal thoracic plates and the lengths of the caudal rami vastly differ between *P*. *echinifer ***n. sp.** and *P*. *cranchii*. Furthermore *P*. *cranchii* does not have slight basal medial expansions on the caudal rami and thus the two species are easily distinguished.

### Molecular analysis

The complete dataset consisted of 585 base pairs belonging to 13 *Pandarus* COI sequences (10 sequences downloaded from Genbank and three newly generated sequences). The uncorrected pairwise sequence divergences estimated amongst the *Pandarus* species range from 0% to 25% (Table 1). The interspecific sequence divergences vary between 15–25% with that between *P*. *satyrus* (PP434798, FJ447391) and *P*. *cranchii* (FJ447387, PP434799, PP434800) being 20–22%, between *P*. *satyrus* (PP434798, FJ447391) and *P*. *smithii* (FJ447388) 16% and 19% and between *P*. *satyrus* (PP434798, FJ447391) and *P*. *sinuatus* (FJ447389, FJ447390) 24 and 25%. The sequence divergence between *P*. *cranchii* (FJ447387, PP434799, PP434800) and *P*. *smithii* (FJ447388) is 22% while that between *P*. *cranchii* (FJ447387, PP434799, PP434800) and *P*. *sinuatus* (FJ447389, FJ447390) are 23 and 24%. The sequence divergence between *P*. *smithii* (FJ447388) and *P*. *sinuatus* (FJ447389, FJ447390) is 21%. Intraspecific sequence divergences vary between 0–3%. The sequence divergence between the two *P*. *satyrus* sequences (collected from *I*. *oxyrhinchus* (FJ447391) and *R*. *typus* (PP434798)) is 3% and the divergence between the two *P*. *sinuatus* sequences (both from *C*. *taurus* (FJ447389, FJ447390)) is 1%. Furthermore, there is no difference between the *P*. *cranchii* sequence collected from *S*. *lewini* (FJ447387) and those collected from *R*. *typus* (morphologically identified as *P*. *cranchii* – PP434799, PP434800) received from Ningaloo Park, Western Australia as well as between the *P*. *cranchii* sequences and the *Pandarus* sp. sequence (KJ551843) also from Ningaloo Reef, Western Australia. Thus, this sequence is also from *P*. *cranchii*. Interestingly, the divergences between the *P*. *cranchii* sequences (FJ447387, KJ551843, PP434799 and PP434800) and that of *P*. *rhincodonicus* (HG942363) (Austin et al. 2016) is only 3%. Thus, *P*. *rhincodonicus* is the same species as *P*. *cranchii* as indicated by a sequence divergence of 0–3%. Furthermore, the sequence divergences between *P*. *satyrus* (PP434798, FJ447391) and others identified as *P*. *satyrus* (collected from *P*. *glauca* from the Mediterranean Sea (OL457303, OL457304, OL457305)) (Palomba et al. 2022) are 15 and 17% falling within the interspecific sequence divergence range rather than the intraspecific sequence divergences. This also applies to the sequence divergences between these *P*. *satyrus* sequences (OL457303, OL457304, OL457305) and all the other sequences in the dataset implying that these sequences may belong to a completely different species.

**Table 1:** Sequence divergences of *Pandarus* specimens using the cytochrome oxidase I gene (585 base pairs).

		1	2	3	4	5	6	7	8	9	10	11	12
1	*P*. *satyrus* PP434798												
2	*P*. *satyrus* FJ447391	0.03											
3	*P*. *cranchii* FJ447387	0.22	0.20										
4	*P*. *cranchii* PP434799	0.22	0.20	0.00									
5	*P*. *cranchii* PP434800	0.22	0.20	0.00	0.00								
6	*Pandarus* sp. KJ551843	0.21	0.19	0.00	0.01	0.00							
7	*P*. *rhincodonicus* HG942363	0.22	0.20	0.03	0.03	0.03	0.03						
8	*P*. *sinuatus* FJ447390	0.24	0.25	0.23	0.24	0.23	0.23	0.23					
9	*P*. *sinuatus* FJ447389	0.24	0.24	0.23	0.23	0.23	0.23	0.22	0.01				
10	*P*. *smithii* FJ447388	0.19	0.16	0.22	0.22	0.22	0.22	0.22	0.21	0.21			
11	*P*. *satyrus* OL457304	0.17	0.15	0.16	0.16	0.16	0.15	0.16	0.24	0.23	0.19		
12	*P*. *satyrus* OL457305	0.17	0.15	0.16	0.16	0.16	0.15	0.16	0.24	0.23	0.19	0.00	
13	*P*. *satyrus* OL457303	0.17	0.15	0.16	0.16	0.16	0.15	0.16	0.24	0.23	0.19	0.00	0.00

Significant non-synonymous substitutions (where more than one sequence share the substitution) in the dataset occurred due to three transitions and one transversion. A first codon position transition is shared by all the *P*. *cranchii* sequences (FJ447387, PP434799, PP434800, KJ551843) and *P*. *rhincodonicus* (HG942363) from GTC/A to ATT at position 94 resulting in a change in the coded amino acid from Valine to Isoleucine. *Pandarus satyrus* (PP434798, FJ447391) and *P*. *sinuatus* (FJ447389, FJ447390) share a change in the coded amino acid from Valine to Isoleucine due to a first codon position transition from GTT/C to ATT at position 328. A transversion at the first codon position 346 from TCT/C to GCT/C resulted in a change in amino acid from Serine to Alanine in *P*. *sinuatus* (FJ447389, FJ447390) and *P*. *smithii* (FJ447388). Lastly a first codon position transition at position 502 from GTT/A to ATC resulted in a change from the amino acid Valine to Isoleusine in *P*. *sinuatus* (FJ447389, FJ447390).

### Remarks

The morphological description of *P*. *rhincodonicus* mentioned distinguishing features such as the dorsal plates of thoracic somite two extending “almost to the limit of the fused plates of” thoracic somite four while the plates of thoracic somite two of *P*. *cranchii* are “considerably shorter” than those of somite four (Norman et al. 2000). However, due to the considerable variation amongst individuals this is not always the case (see Fig. 2a) and additionally, *P*. *satyrus* also have plates of thoracic somite two almost as long as those of somite four (see Fig. 3b, Fig. 1 in Cressey (1967)). Additionally, “the posterior margin of the cephalon is armed with four tubercules instead of the serrated margin” (Norman et al. 2000) is also seen in other species e.g. *P*. *cranchii* (see Izawa 2010), *P*. *satyrus* (see Fig. 3a), *P*. *sinuatus* (see Fig. 7a) and *P*. *echinifer* **n. sp.** (see Fig. 10a) while that of *P*. *smithii* seems more serrated (see Fig. 5a, Fig. 7a in Izawa (2010)). “The shape of the caudal rami differ as they lack the large inner lobe characteristic of *P*. *cranchii*” (Norman et al. 2000) is invalid since *P*. *cranchii* do not have caudal rami with basal medial expansions (see Figs. A, D in Izawa (2010)). Thus, no valid distinguishing morphological characteristics are found for *P*. *rhincodonicus* while the description included features applicable to both *P*. *cranchii* and *P*. *satyrus* (see Fig. 1A in Norman et al. (2000)). It is therefore suspected that *P*. *rhincodonicus* is a synonym of *P*. *cranchii*. Based on evidence from the molecular analysis which included the sequence of *P*. *rhincodonicus*, downloaded from Genbank (and others collected from *R*. *typus* at Ningaloo Park and morphologically identified as *P*. *cranchii*) indicated that the maximum sequence divergence amongst these sequences is 3% (Table 1) similar to expected intraspecific variation and thus *P*. *rhincodonicus* is a synonym of *P*. *cranchii*.

## Discussion

Of the current 14 valid species of *Pandarus* (Walter & Boxshall 2024), *P*. *rouxii*, *P*. *brevicaudis* and *P*. *ambiguus* should be considered as *species inquirenda*. Additionally, *P*. *rhincodonicus* is a synonym of *P*. *cranchii* and *P*. *echinifer* **n. sp.** is newly described. Thus, the number of valid *Pandarus* species currently is 11.

The identification of *Pandarus* species is compromised due to different *Pandarus* species that share many characteristics e.g. the relative lengths of the dorsal thoracic plates as well as varying signs of pigmentation amongst individuals (Ho 1963; Cressey 1967; Kabata 1979) and the same spine and setal formulas (Cressey 1967), while different individuals of the same species exhibit variation in the setation of the limbs (Hewitt 1967). Even though the division into the “bicolor” and “cranchii” groups can still be used as a starting point, researchers should be careful as in some individuals this is not absolutely clear (see Fig. 7a) as is the presence of the basal medial expansions to distinguish *P*. *smithii* (*cf*. Figs. 5b, 6a, 8a, 10b). Additionally, different species are symbionts of several host species (see current study, Shiino (1954), Lewis (1966), Cressey (1967), Alvarez & Winfield (2001) and Izawa (2010)).

From the current study, it seems like the detailed structure of the distomedial spine on the last segment of the exopod of leg 1 may be a distinguishing feature amongst similar *Pandarus* species (*cf*. Figs. 2c, d, 4f, 6b–d, 8d–f, 9a, 11b, 12a, b). Even though the distomedial spine is quite small, differences can be observed using light microscopy (see Figs. 2d, 6d, 8f, 12a, b). This character may be specifically useful if used in combination with other less distinguishing features such as the relative lengths of the dorsal plates and the caudal rami.

This report constitutes the first record of *P*. *bicolor* from *M*. *mustelus* off the west coast (Atlantic Ocean) SA while it is also the first record of *P*. *cranchii* from *R*. *typus* off the west coast (Indian Ocean) Australia. Furthermore, it is the first report of *P*. *satyrus* from *C*. *carcharias*, *I*. *oxyrinchus*, *C*. *taurus* off the east coast (Indian Ocean) SA as well as from *R*. *typus* off the west coast (Atlantic Ocean) SA and the west coast (Indian Ocean) Australia. Additionally, *P*. *smithii* is for the first time reported from *C*. *brevipinna* off the east coast (Indian Ocean) SA as well as from *R*. *typus* off the west coast (Atlantic Ocean) SA.
